# Mesenchymal Stem Cells Stabilize Atherosclerotic Vulnerable Plaque by Anti-Inflammatory Properties

**DOI:** 10.1371/journal.pone.0136026

**Published:** 2015-08-19

**Authors:** Shuang-shuang Wang, Si-wang Hu, Qing-hua Zhang, Ai-xiang Xia, Zhi-xin Jiang, Xiao-min Chen

**Affiliations:** 1 College of Medicine, Zhejiang University, Hangzhou, 310009, China; 2 Department of Cardiology, Ningbo First Hospital, Ningbo, 315000, China; 3 Department of Spine Surgery, Affiliated Hospital of Medical College of Ningbo University, Ningbo 315000, China; 4 Chinese PLA 305 hospital, Beijing 100017, China; Zhongshan Hospital Fudan University, CHINA

## Abstract

**Background and objectives:**

Formation and progression of atherosclerotic vulnerable plaque (VP) is the primary cause of many cardio-cerebrovascular diseases such as acute coronary syndrome and stroke. It has been reported that bone marrow mesenchymal stem cells (MSC) exhibit protective effects against many kinds of diseases including myocardial infarction. Here, we examined the effects of intravenous MSC infusion on a VP model and provide novel evidence of its influence as a therapy in this animal disease model.

**Subjects and methods:**

Thirty healthy male New Zealand white rabbits were randomly divided into a MSC, VP or stable plaque (SP) group (n = 10/group) and received high fat diet and cold-induced common carotid artery intimal injury with liquid nitrogen to form atherosclerotic plaques. Serum hs-CRP, TNF-α, IL-6 and IL-10 levels were measured by ELISA at 1, 2, 3, 7, 14, 21 and 28 days after MSC transplantation. The animals were sacrificed at 4 weeks after MSC transplantation. Lesions in the right common carotid were observed using H&E and Masson staining, and the fibrous cap/lipid core ratio of atherosclerotic plaques were calculated. The expression of nuclear factor κB (NF-κB) and matrix metalloproteinase 1, 2, 9 (MMP-1,2,9) in the plaque were detected using immunohistochemistry, and apoptotic cells in the plaques were detected by TUNEL. In addition, the level of TNF-α stimulated gene/protein 6 (TSG-6) mRNA and protein were measured by quantitative Real-Time PCR and Western blotting, respectively.

**Results:**

Two rabbits in the VP group died of lung infection and cerebral infarction respectively at 1 week after plaque injury by liquid nitrogen. Both H&E and Masson staining revealed that the plaques from the SP and MSC groups had more stable morphological structure and a larger fibrous cap/lipid core ratio than the VP group. Serum hs-CRP, TNF-α and IL-6 were significantly down-regulated, whereas IL-10 was significantly up-regulated in the MSC group compared with the VP group. .Immunohistochemistry analysis revealed that NF-κB and MMP expression was reduced in the MSC and SP groups compared to the VP group. Cell apoptosis decreased significantly in both the MSC and SP groups in comparison to the VP group. TSG-6 mRNA and protein expression were higher in the plaques of the MSC group compared to the VP and SP groups.

**Conclusions:**

Our study results suggest that MSC transplantation can effectively stabilize vulnerable plaques in atherosclerotic rabbits. This may potentially offer a new clinical application of MSC in atherosclerosis.

## Introduction

Rupture of atherosclerotic plaques and the ensuing occlusive thrombus formation play important roles in the onset of two major causes of death worldwide: acute coronary syndrome (ACS) and ischemic stroke [1,2]. Although atherosclerotic plaque formation is a complex multifaceted process, inflammation has been identified as a critical underlying factor [3]. In fact, a major predictor of plaque instability is a continuing inflammatory response within the atherosclerotic plaque. Inflammatory cells such as mononuclear macrophages and T lymphocytes infiltrate vulnerable plaques (VP), which can promote production and release of a variety of inflammatory mediators including interleukins (IL) and tumor necrosis factor-α (TNF-α). These inflammatory mediators directly and indirectly act on neighboring cells and stromal elements resulting in changes in plaque morphology, consequently making it more susceptible to rupture [4].

Cell-based treatments have gained recent interest and bone marrow mesenchymal stem cell (MSC) therapy is strongly emerging as a potentially viable cell therapy. MSCs are widely present in adult tissues and can proliferate and differentiate along multiple lineages giving rise to muscle, brain, liver, cartilage, bone, fat and blood vessel *in vitro* and *in vivo* [5,6]. MSC transplantation has been shown to be efficacious in a variety of diseases such as myocardial infarction, corneal damage, lung injury, and its efficacy has, in part, been attributed to its anti-inflammatory properties [7–9]. In addition, MSCs exhibit many other important biological actions including: (1) ability to colonize damaged tissue and subsequently differentiate into cells important for tissue repair, (2) immuno-modulatory effects through paracrine/autocrine signaling such as inhibition of cytotoxic T lymphocytes and natural killer cells that lessens the immune response within diseased tissue, (3) hematopoietic support functions and (4) secretion of various bioactive substances. Many clinical and experimental studies have substantiated these biological functions of MSCs cultured *in vitro*.

However, to date there is little known about the anti-inflammatory properties of implanted MSCs on atherosclerotic vulnerable plaques. To address this fundamental gap in our knowledge, we used a rabbit model of atherosclerotic vulnerable plaque to explore the effects of engrafted MSCs on various indicators of inflammation including serum inflammatory mediator levels at various time points following transplantation, plaque morphology, expression of nuclear factor-κB (NF-κB), matrixmetalloproteinases (MMPs) and TSG-6, and apoptotic cell number in the plaques. Overall, we found that MSC transplantation reversed the inflammatory response seen with vulnerable plaque and enhanced anti-inflammatory effects demonstrating greater plaque stability with transplantation of MSCs.

## Methods

### Animals

Two month old New Zealand white rabbits (weight, 2.3±0.5 kg) were obtained from and housed in the Laboratorial Animal Center of the People’s Hospital of Peking University, China. All animals were monitored and cared for by animal care staff twice daily. Animal care and surgical operations were performed in adherence to (Beijing Administration Guidelines for the Use of of Laboratory Animals) and the animal research protocol was approved by the Ethics Committee for the Use of Human or Animal Subjects of Chinese PLA 305 Hospital (Permit Number: 2010–06). All surgerical procedures were performed under approved general anesthesia, and after procedure, the animals received ibuprofen in their water for 3 days for pain relief. All efforts were made to minimize animal suffering during the study.

### Atherosclerotic vulnerable plaque model

**Fig 1:**
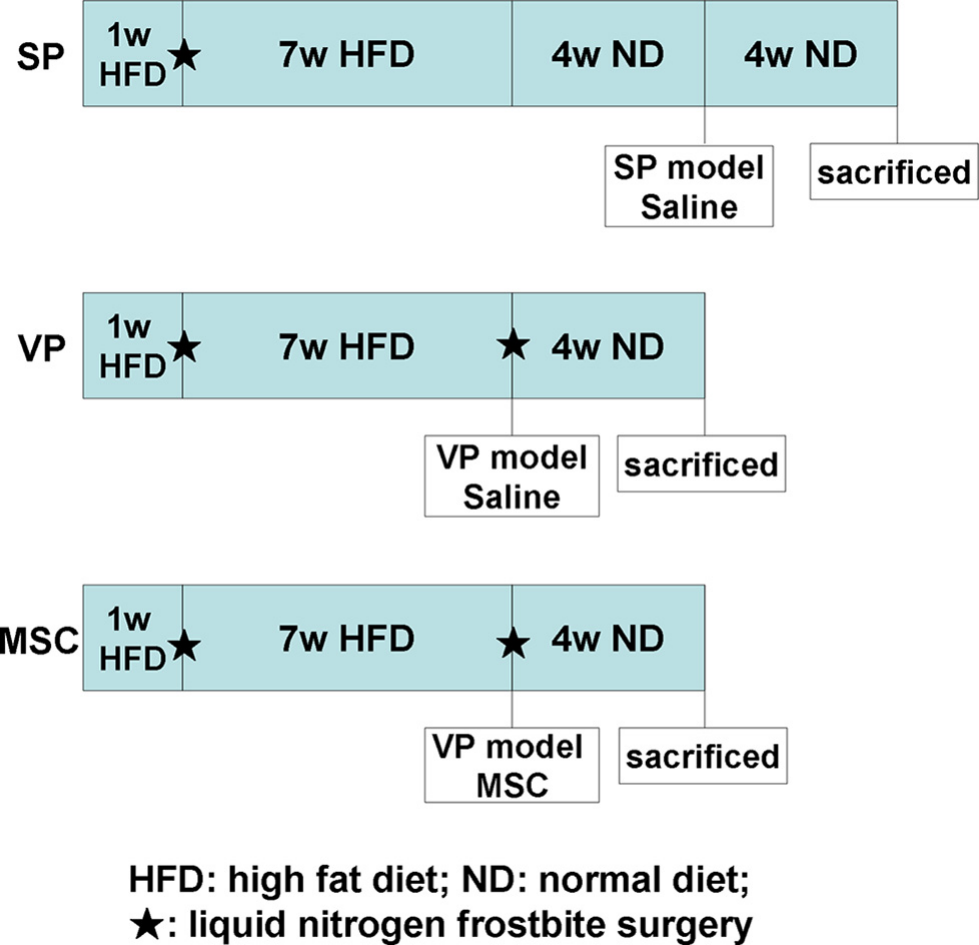
The treatments on animals in different groups. Rabbits were maintained on a high fat diet (HFD) for one week after which they underwent carotid artery intimal injury with liquid nitrogen to induce atherosclerotic plaques. Rabbits were continued on a high fat diet for an additional 7 weeks for plaque formation. To induce vulnerable plaques, another cold-induced intimal injury was performed on rabbits of VP and MSC groups. Rabbits in SP group accepted a normal diet for 4 weeks instead of the second intimal injury to stabilize plaques. HFD: high fat diet; ND: normal diet; ★: cold-induced common carotid artery intimal injury with liquid nitrogen.

Atherosclerotic vulnerable plaque formation was induced in rabbits as described previously [10]. Briefly, to induce atherosclerotic plaques, the rabbits were maintained on a high fat diet (1% cholesterol, 3% lard, and 15% yolk) for 1 week after which they underwent cold-induced common carotid artery intimal injury with liquid nitrogen under general anesthesia with intravenous injection of pentobarbital sodium (30 mg/kg). Rabbits were continued on a high fat diet for an additional 7 weeks for plaque formation. And then another cold-induced intimal injury was performed to induce vulnerable plaques. For stable plaque formation, the rabbits were maintained on a high fat diet for 1 week followed by a liquid nitrogen frostbite surgery. Rabbits continued a high fat diet for 7 weeks after surgery followed by a normal diet for 4 weeks. In total, 30 rabbits were randomly divided into three groups and received the following treatment: 1) vulnerable plaque and 1 mL saline (VP group, n = 10), 2) vulnerable plaque and 1 × 10^7^ MSCs (MSC group, n = 10), and 3) stable plaque and 1 mL saline (SP group, n = 10). At the end of the study, the animals were sacrificed with intravenous injection a lethal dose of pentobarbital sodium (Fig 1).

### Cold-induced common carotid artery intimal injury with liquid nitrogen

With the rabbit under sufficient general anesthesia, a midline incision was made on the neck and the right carotid artery was surgically exposed. A 4-cm segment of artery was isolated by two artery clamps. A 1-ml aseptic syringe needle was bent upwards to form an 45°angle and inserted into the proximal end of the segment. Blood was rinsed from the segment with saline, and the segment was evacuated completely, then liquid nitrogen (about 1 ml) was injected as quickly as possible into the empty artery through a 5-ml syringe via the bent and indwelling needle. To ensure intimal injury of the carotid artery, this process was repeated three times in five minutes. The segment was then rinsed with saline, the needle was withdrawn, and the artery clamps were loosened from the distal end to the proximal end. Circulation was re-established, hemostasis was ensured, and the surgical incision was closed.

### Marrow harvesting

One-month old New Zealand white rabbits (weight, 0.8–1.0kg) were used for marrow harvesting. Under general anesthesia (intramuscular injection ketamine hydrochloride [5 mg/kg] and Sumianxin II [5 mg/kg]), the proximal tibia was prepared using hair removal cream and subsequently disinfected, and approximately 10 mL of bone marrow was aspirated out using a medullo-puncture needle in a heparinized syringe.

### Isolation, expansion and characterization of MSCs

Mononuclear cells were isolated by gradient centrifugation at 900 g for 30 min in Percoll (Sigma, St Louis, Missouri) at a density of 1.073 g/mL. The cells were then washed twice with phosphate-buffered saline (PBS). Cells were plated at a density of 1 × 10^6^ cells in 25 cm^2^ culture flasks containing 5 mL of complete culture medium: DMEM/F12 (1:1) (Hyclone, Logan, UT), 15% fetal bovine serum (Gibco, Grand Island, NY), 100 U/mL penicillin and 100 μg/mL streptomycin (Gibco). Cells were maintained at 37°C with 5% CO_2_. After three days *in vitro*, non-adherent cells were removed by replacing media with fresh culture medium. When the cells reached 80% to 90% confluence, adherent cells were trypsinized, harvested and expanded. Expanded cells from passages three to eight were used for transplantation. MSCs were assessed by flow cytometry analysis for the markers CD29 (Abcam, Cambridge, MA), CD44 (AbD Serotec, Kidlington, UK), CD90 (Abcam), CD14 (Abcam) and CD45 (AbD Serotec) according to standard protocols [11].

### MSC transplantation

After the second intimal injury, MSCs (1 × 10^7^ cells) or vehicle (PBS) was intravenously administered via the ear vein. The SP group of rabbits also received PBS administration after four weeks of normal diet.

### Enzyme-linked immunosorbent assay (ELISA)

Blood was collected from the ear vein on days 1, 3, 7, 14, 21 and 28 post-MSC injection. Serum hs-CRP, TNF-α, IL-6 and IL-10 levels were measured using an Enzyme Linked Immunosorbent Assay (ELISA) kit (Bluegene biotech, P.R. China) according to the manufacturers' instructions. ELISA experiments were performed in triplicate.

### Histological analysis and immunohistochemistry

The common carotid arteries were excised and perfused with phosphate-buffered saline on day 28 post-MSC injection and fixed with 4% paraformaldehyde for 24 hrs at room temperature. Next the arteries were embedded in paraffin, sectioned at 4 μm thickness and stained with either hematoxylin and eosin (H&E) or Masson's trichrome in accordance with standard procedures. Light microscopy was used to observe the morphology of plaques and the Image-Pro Plus Image analysis system was used to acquire and analyze histologic images.

Paraffin-embedded artery sections were exposed to increasing ethanol concentrations followed by a PBS wash. Sections were incubated with Protein Block (Boster) with specific rabbit polyclonal antibodies against NF-κB(p65), MMP-1, MMP-2, and MMP-9 (Abcam) in diluent for 40 min followed by incubation with corresponding secondary antibodies and PAP (peroxidase–anti-peroxidase) complex. Lastly, the sections were visualized using DAB (3,39-diaminobenzidine). The grey values of the positive area in the plaques were evaluated at x400 magnification using Image J in 5 randomly selected fields on each slide by an independent experienced pathologist blinded to the treatment conditions.

### TUNEL staining

TUNEL assay was performed to determine the number of apoptotic cells in atherosclerotic plaques. TUNEL staining was performed according to the manufacturer’s instructions (Roche Molecular Biochemicals, Swiss). Healthy cells showed light blue staining within the nucleus while apoptotic cells exhibited brown and/or dark blue nucleus staining. A minimum of 200 cells were counted on each slide. The TUNEL-positive cells that also showed apoptotic nuclear morphology were defined as apoptotic, and the percentage of these cells to the total cell number was recorded as an apoptotic index.

### Quantitative real-time polymerase chain reaction

Total RNA was extracted from the common carotid artery at 28 days after MSC transplantation using TRIzol Reagent (Invitrogen Corp, Carlsbad, CA) according to the manufacturer’s instructions. Levels of TNF-α stimulated gene/protein 6 (TSG-6) mRNA were quantitated using an ABI 7500HT Fast Real-Time PCR System (Applied Biosystems, Grand Island, NY, USA). Glyceraldehyde-3-phosphate dehydrogenase (GAPDH) was used as an endogenous control. Sequence-specific primers for the above genes were designed using Premier 5 software as follows (Table 1).

**Table 1:** Primer sequences used in real-time PCR.

GENE		Sequences(5’——3’)
TSG-6	Forward	TTGTGAAGCCAGGGTCCAAT
	Reverse	GTTGTAGCAATAGGCATCCCATCT
GAPDH	Forward	GGACCAGGTTGTCTCCTGTG
	Reverse	TGTAGGCCATGAGGTCCAC

The 2-ΔΔCt method was used to calculate relative expression levels.

### Western blotting

Common carotid artery tissue was prepared using lysis buffer with protease inhibitors (1 mmol/L benzamidine, 1 lg/mL leupeptin, 10 lg/mL soybean trypsin inhibitor, and 0.5 mmol/L PMSF), according to the manufacturer’s instructions. Samples were centrifuged at 8000 rpm for 15 min and the supernatant was collected. Total protein content of the samples was measured using the Bradford method, and sample aliquots containing equal amounts of protein were boiled for 5 min in sample loading buffer and separated by sodium dodecylsulphate polyacrylamide gel electrophoresis (SDS-PAGE) followed by transfer onto PVDF membranes (Immobilon-P, Millipore). Membranes were subsequently blocked with 5% non-fat dry milk in TBS containing 0.1% Tween 20 (TBST). Next, PVDF membranes were incubated with the primary antibodies anti-TSG6 (1:500), or anti-β-actin (1:3,000 diluted in TBS-T) overnight at 4°C, followed by an appropriate secondary antibody (horseradish peroxidase-conjugated anti-goat IgG) for 1 h at room temperature. The reaction was visualized by enhanced chemiluminescence (ECL). Band intensities were measured by ImageJ software (Wayne Rasband, NIH) and densitometry values were normalized to that of β-actin.

### Statistical analysis

Data were expressed as mean ± standard error (S.E.). Comparisons of parameters among groups were made by one-way ANOVA and repeated measures ANOVA, followed by Newman–Keuls' post-hoc test. Differences were considered statistically significant when P < 0.05.

## Results

### Isolation and characterization of MSCs

**Fig 2:**
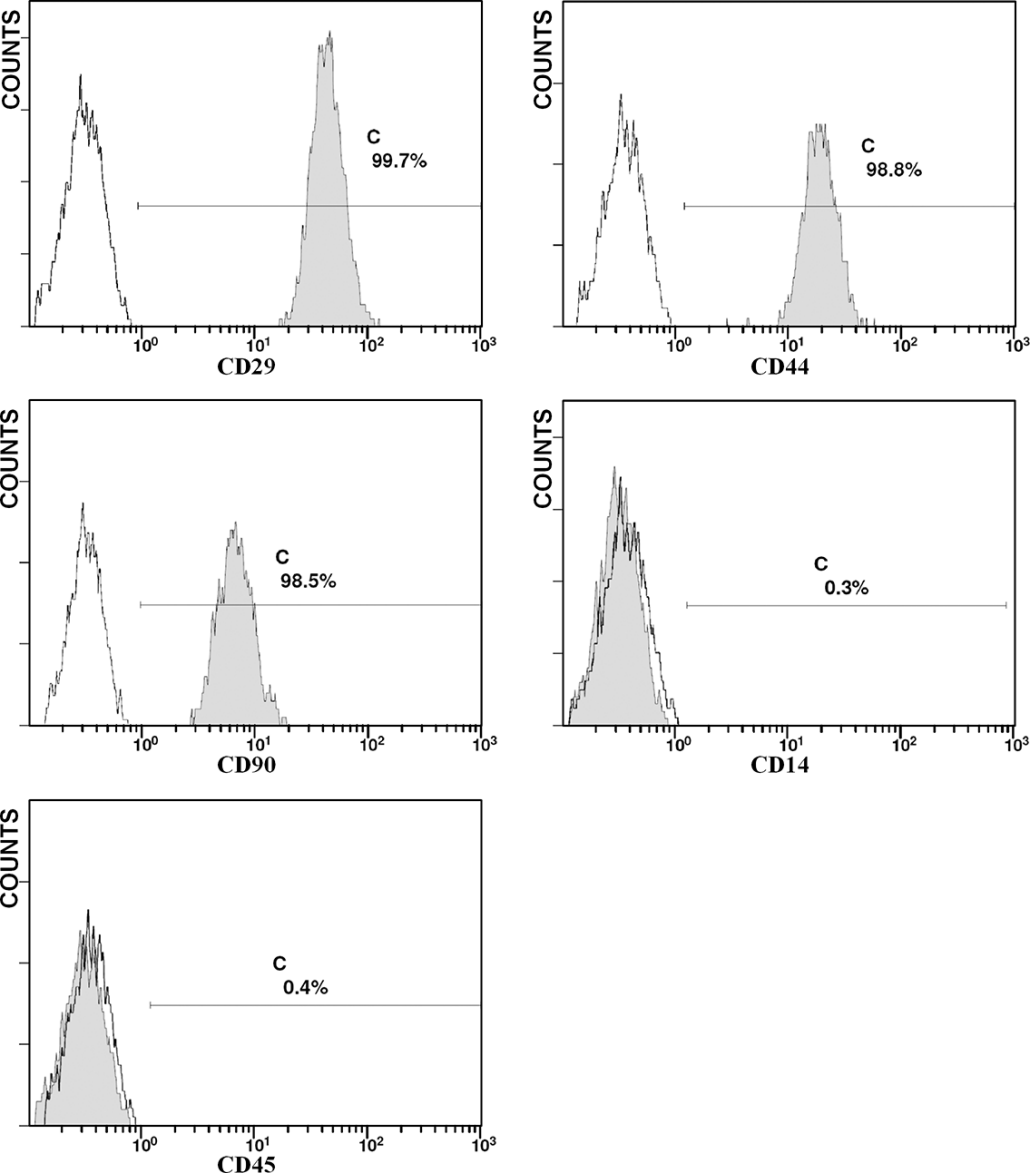
Surface marker expression in MSCs. Confirmation of MSC identity by flow cytometry analysis. Cells were positive for CD29 (99.7%), CD44 (98.8%) and CD90 (98.5%) and showed low expression of CD14 (0.3%), and CD45 (0.4%). The black lines are isotype controls.

MSCs were isolated from the bone marrow of rabbits and maintained in culture for several passages. They displayed fibroblast-like cell morphology and formed homogenous colonies. MSCs do not exhibit a specific antigen profile. At a minimum, MSCs should express stem cell markers such as CD105, CD90, CD73, and CD29, and always be positive for CD44 and negative for CD14 and CD45 [12–14]. Before intravenous transplantation, third passage cells were characterized. Flow cytometry analysis confirmed that the cells were positive for CD29 (99.7%), CD44 (98.8%) and CD90 (98.5%), and they had low expression of CD14 (0.3%), and CD45 (0.4%) demonstrating stem cell like properties (Fig 2). MSCs in our study closely conformed to the characteristics of MSCs.

### Animal response to plaque induction

After the plaques were induced by liquid nitrogen, one rabbit in the VP group developed symptoms of cough and lack of appetite, and died from lung infection after 5 days’ penicillin treatment. One animal in the VP group developed cerebral infarction and showed symptoms of hemiparalysis, and was humanely euthanized by a lethal dose of pentobarbital sodium. The other 28 rabbits completed this experiment (10 in the MSC group, 8 in the VP group, and 10 in the SP group).

### Serum levels of pro- and anti- inflammatory cytokines

**Fig 3:**
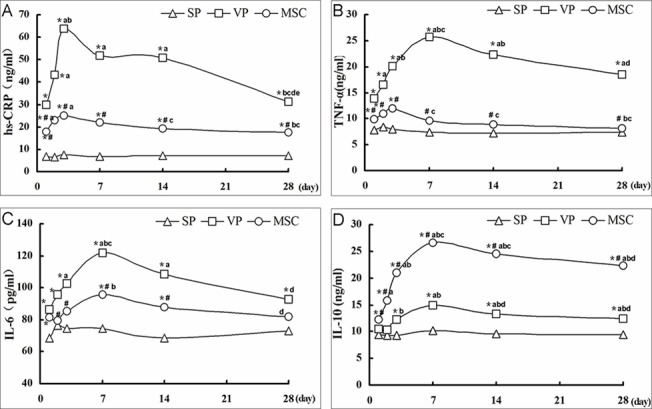
Effect of MSC transplantation on serum hs-CRP, TNF-α, IL-6 and IL-10 levels. Cytokines were analyzed by ELISA on days 1, 2, 3, 7, 14 and 28 after MSC or vehicle injection. The levels of hs-CRP, TNF-α, IL-6 and IL-10 in the SP group was relatively steady (P>0.05). Compared to the SP group, hs-CRP, TNF-α and IL-6 were significantly increased in the VP group from 1d to 28d. All three cytokines were also higher in the MSC group compared to the SP group except for TNF-α on 7–28 d; IL-6 on 2, 3 and 28 days; and IL-10 on 1–2 d. MSCs significantly reduced the levels of these three cytokines compared with the VP group (P < 0.05), except IL-6 on 1d and 28 d (A,B,C). After plaque induction, IL-10 was significantly higher in the VP group than SP group from 3–28 d. MSC transplantation further increased IL-10 from 1 to 28d compared with the SP and VP groups (P < 0.05) (D). *,compared with SP group, P < 0.05; #, compared with VP group, P < 0.05; a, comparison with 1 d, P < 0.05; b, compared with 2 d, P < 0.05; c, compared with 3 d, P < 0.05; d, compared with 7 d, P < 0.05; e, compared with 14 d, P < 0.05.

To investigate the effects of MSC transplantation on the inflammatory response, we measured serum cytokine levels in the three experimental groups. Hs-CRP, TNF-α and IL-6 are pro-inflammatory cytokines that serve as important serum markers, reflecting the body's inflammatory state and predicting future coronary events in patients with stable and unstable disease [15–18]. Levels of all three cytokines increased after plaque formation was induced (VP group), and hs-CRP peaked at 3 d while TNF-α and IL-6 peaked at 7 d. MSC transplantation significantly reduced the levels of all three cytokines compared to the VP group (P < 0.05) with the exception of IL-6 at 1 and 28 d. We also examined IL-10 levels, an anti-inflammatory cytokine, that can reduce tissue inflammation after injury [18]. After plaque induction, IL-10 levels were significantly higher in the VP group than in the SP group from 3–28 d. The MSC group had an even greater increase in IL-10 from 1–28 d compared with the SP and VP groups (P < 0.05). Hs-CRP had no significant differences at different time points in the SP group (P>0.05), which was consistent with TNF-α, IL-6 and IL-10 (Fig 3, Tables A-D in S1 File). These results indicated that MSC transplantation decreased the pathological inflammatory response by reducing the levels of circulating pro-inflammatory cytokines hs-CRP, TNF-α and IL-6, while increasing the anti-inflammatory cytokine, IL-10.

In addition, we found that hs-CRP, TNF-α, IL-6 and IL-10 levels exhibited significant differences at different time points, with an initial elevation followed by a gradual decline in expression. The variation trends of hs-CRP, TNF-α and IL-6 at different times were different. Hs-CRP was expressed highest at the 3rd day, and the greatest reduction was observed at the 1st week followed by a slower deacrease. TNF-α, IL-6 and IL-10 peaked in the first week after transplantation and then gradually declined. This may have resulted because the different factors had different reaction times and different secretion peaks, which agrees with prior studies.

### Plaque morphology in carotid arteries

**Fig 4:**
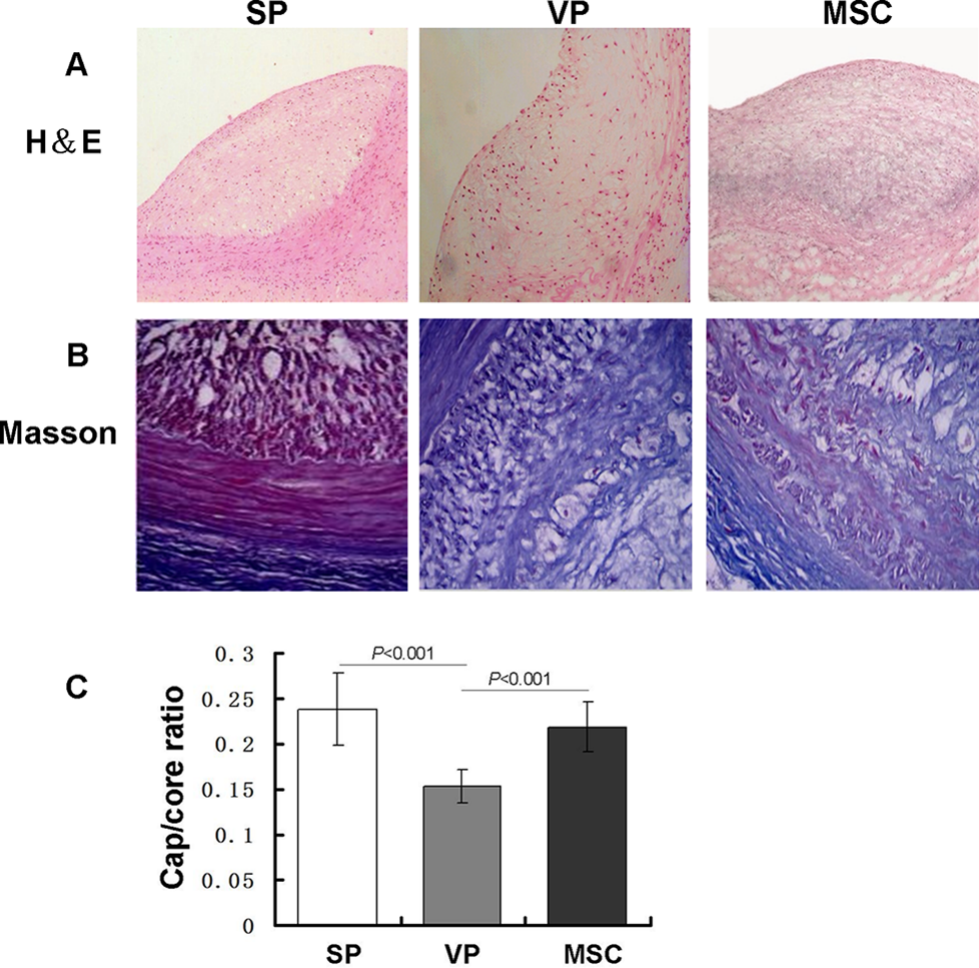
H&E and Massons trichome stained plaques and cap/core ratio. Morphology and cap/core ratios of atherosclerotic plaques in the SP, VP and MSC groups. (A) shows H&E stained plaque (100×). The SP group plaque had intact morphological structure with a thick fibrous cap and minimal inflammatory cells. In the VP group, the plaque had massive lipid cores covered by a thin fibrous cap with a large number of inflammatory cells infiltrated and some plaques ruptured and/or clotted. The plaque structure in the MSC group was intermediate. (B) shows Masson’s trichome stained smooth muscle cells and collagen fibers in the plaque of all three groups. The blue represent collagen fibers. Red represent smooth muscle cells (400×). SP group plaques contained a large number of smooth muscle cells and collagen fibers while the MSC group had the next greatest amount. Compared with the MSC and SP group, the VP group plaque had a decreased and disordered arrangement of smooth muscle cells and collagen. (C) shows cap/core ratios of all three groups. The fibrous cap/lipid core ratio of atherosclerotic plaque was higher in the SP group (0.238±0.040) and the MSC group (0.219±0.027) than in the VP group (0.153±0.018) (P<0.001, P<0.001).

To investigate the pathology of atherosclerotic plaque, sections of the common carotid arteries from each of the three groups were stained using H&E and Masson’s stain. Fig 4A shows representative histological images from the three groups at 28 d after MSC/vehicle injection. Plaques in the SP group had a thick fibrous cap with an intact morphological structure with no evidence of ruptured plaque and a small number of inflammatory cells. In the VP group, plaques contained massive lipid cores and were covered by a thin fibrous cap with residual foam cells present and a large number of inflitrated inflammatory cells (including macrophages and lymphocytes). Morphology of plaque in the MSC group had characteristics of both the VP and SP groups. The fibrous cap/lipid core ratio of atherosclerotic plaque was higher in MSC group (0.219±0.027) and SP group (0.238±0.040) compared to the VP group (0.153±0.018) (P<0.01) (Fig 4C, Table E in S1 File). The Masson staining revealed that plaques in the SP group contained a large number of smooth muscle cells and elastic fibers, as did the MSC group. Compared with the MSC and SP groups, the VP group plaque had decreased smooth muscle cells and elastic fibers and had a disordered arrangement of collagen fibers (Fig 4B). These histological findings indicate the morphological structure of atherosclerotic vulnerable plaque became more stable after MSC transplantation.

### Protein expression levels of NF-κB and MMPs in plaques

**Fig 5:**
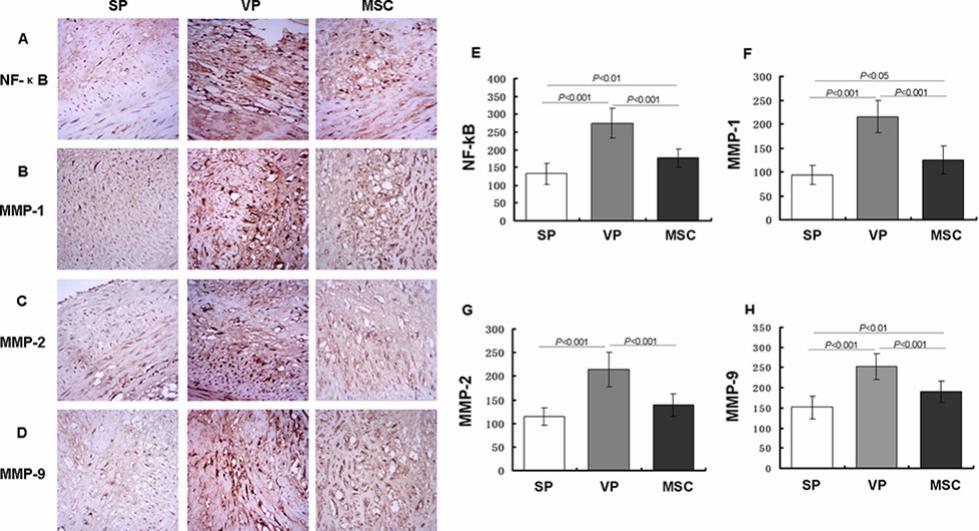
Influence of MSCs on NF-κB and MMPs in plaques. The NF-κB, MMP-1, MMP-2 and MMP-9 showed a low expression in SP group, and compared with SP group, the VP group displayed higher expression (P<0.001, respectively). Compared with the VP group, the MSC group displayed significantly decreased NF-κB, MMP-1, MMP-2 and MMP-9 levels (P< 0.001, respectively). The levels of NF-κB, MMP-1 and MMP-9 in MSC group were markedly higher than the SP group (P<0.01, P<0.05, P<0.01, respectively).

To further explore the effects of MSC transplantation on the inflammatory reaction in the plaques, we measured the expression levels of NF-κB, MMP-1, MMP-2 and MMP-9. NF-κB is a key transcription factor regulating the expression of factors involved in the inflammatory response. MMP-1, MMP-2 and MMP-9 increase the metabolism of collagen, and their expression and activity correlate strongly with plaque stability [19,20]. The SP group had a low expression of NF-κB, MMP-1, MMP-2 and MMP-9 compared to the VP group (P < 0.001). Compared with the VP group, the MSC group displayed markedly decreased NF-κB, MMP-1, MMP-2 and MMP-9 levels (P < 0.001) (Fig 5, Table F in S1 File). Overall, MSC transplantation reduced the expression of NF-κB, MMP1, MMP-2 and MMP-9 in atherosclerotic plaque, which could potentially increase plaque stability.

### Apoptotic cells in plaques

**Fig 6:**
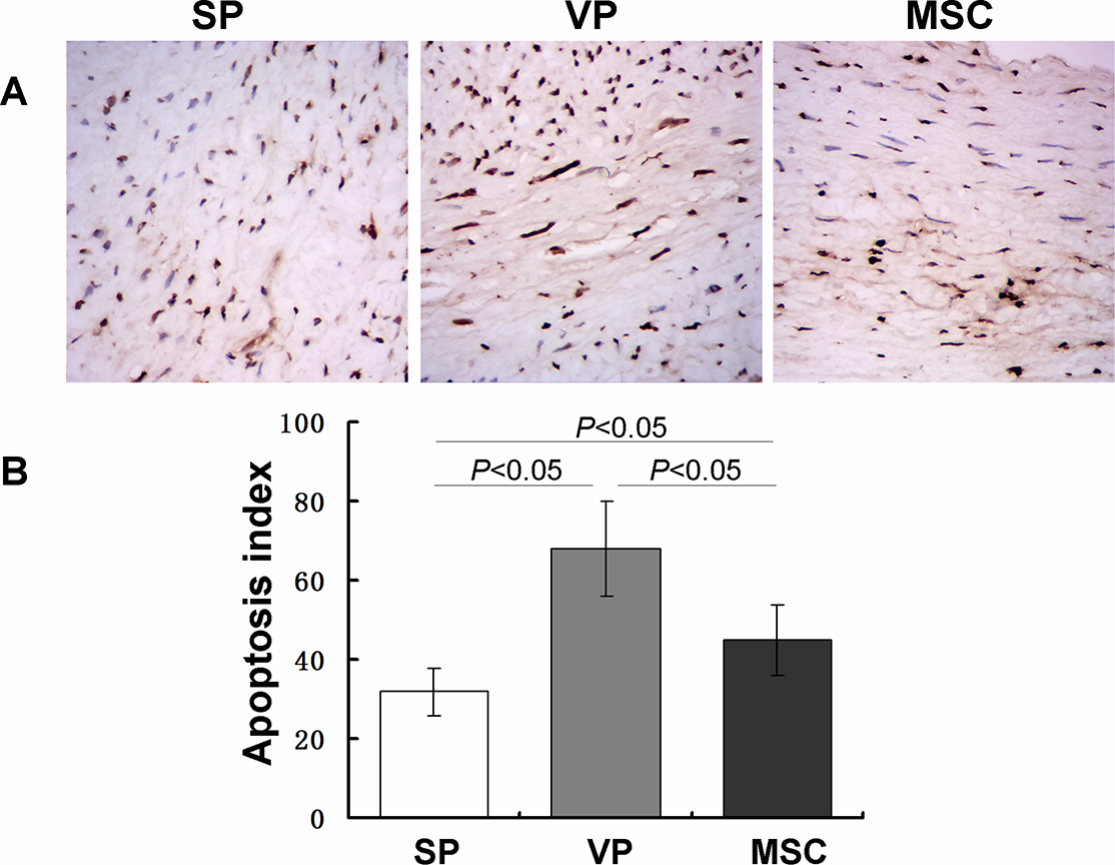
Influence of MSC administration on apoptotic cells in atherosclerotic plaques. Apoptotic cells in the MSC and VP groups were mainly distributed in the lipid core and tunica media with few in the intima. However, in the SP group apoptotic cells were mainly located in the tunica media though a small number were present in the lipid core and intima (A). The number of apoptotic cells in the VP group plaques were significantly increased compared with the SP group (68.08±12.00 VS 32.67±5.85) (P < 0.05). After MSC transplantation, apoptotic cells decreased markedly compared with the VP group (45.17±9.66 VS 68.08±12.00) (P < 0.05). Furthermore, the apoptotic cells in plaques of the MSC group were higher than that in the SP group.

From our H&E and Masson’s staining, it appeared that the number of apoptotic cells varied between groups; therefore we used TUNEL staining to quantify the number of apoptotic cells in the three groups. We found that the number of apoptotic cells present in plaques in the VP group was significantly higher than in the SP group (P < 0.05) (Fig 6, Table G in S1 File). The MSC group showed a significant decrease in the number of apoptotic cells in plaques compared with the VP group (P < 0.05). These data clearly indicate that MSCs inhibited cell apoptosis in vulnerable plaques.

### mRNA and protein expression of TSG-6 in carotid arteries

**Fig 7:**
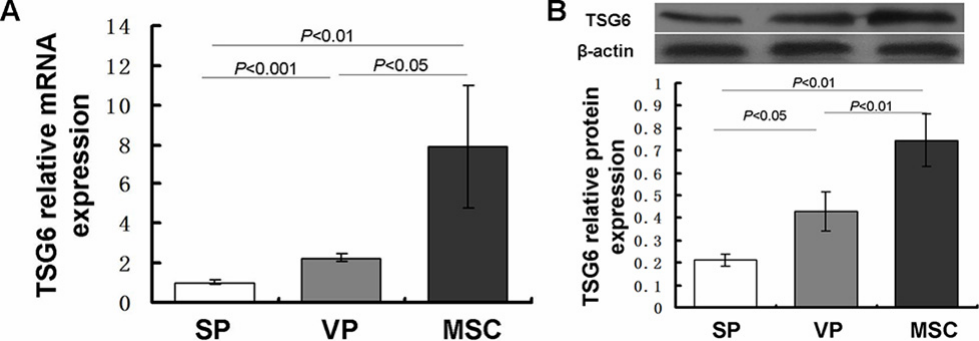
TSG-6 mRNA and protein expression evaluated by Real-time PCR and Western blot. TSG-6 mRNA levels were compared to GAPDH mRNA and TSG-6 protein levels were compared to β-actin protein values and are expressed as mean±SD. TSG6 mRNA expression in the VP group (2.27±0.18) was markedly higher than the SP group (1.01±0.12) (P < 0.001). TSG-6 in the MSC group (7.89±3.08) was significantly higher than both the VP and SP groups, respectively (P < 0.05, P < 0.01) (A). TSG-6 protein expression was similarly up-regulated in the plaque of the MSC (0.747±0.107) and VP groups (0.409±0.102) compared to the SP group (0.226±0.059) (P < 0.001, P < 0.01). TSG-6 in the MSC group was further increased compared to the VP group (P < 0.01) (B).

We next analyzed the expression of the anti-inflammatory factor TSG-6. TSG-6 plays an important role in the inflammatory process due to its anti-inflammatory effects. We found that both TSG-6 mRNA and protein were up-regulated after MSC transplantation compared with the VP and SP groups (P < 0.001) (Fig 7, Table H in S1 File). These results revealed that MSC transplantation increased RNA and protein expression of TSG-6 in atherosclerotic plaque.

## Discussion

Cardiovascular and cerebrovascular diseases, including myocardial infarction and stroke, are the most frequent causes of morbidity and mortality in the world and represent a central challenge for modern research and medicine. Rupture and thrombo-occlusion of the atherosclerotic plaque accounts for approximately 70% of fatal acute myocardial infarctions and/or sudden coronary deaths [1]. Clinical risk is determined by assessing the stability of atherosclerotic plaques rather than the size of the plaques. It is generally accepted that plaques with a thin fibrous cap, large lipid core and a large number of mononuclear macrophages are unstable and prone to rupturing, which can lead to the occurrence of acute cardio-cerebrovascular events. Atherosclerosis has been suggested by many to be a nonlinear process whereby plaques can alternate between stable and vulnerable states according to the changes of internal environment. However, the inflammatory response persists throughout the development of atherosclerosis and is thus considered a critical factor in the development of vulnerable plaques.

Bone marrow–derived mesenchymal stem cells have received a lot of attention for their efficacy as a medical therapeutic strategy. MSCs have a wide range of advantages compared with other stem cell populations including their low immunogenicity, multi-differentiation characteristics, easy cultivation and amplification *in vitro*, and a unique “homing” feature following transplantation. Recently, the therapeutic benefits of intravenous or local MSC transplantation have been observed in many diseases and injury models including acute lung injury [9,21], myocardial infarction [22], acute renal failure [23], cerebral ischemia [24], Alzheimer’s disease [25] and corneal damage [26]. These studies highlight the feasibility of using MSCs, as well as the anti-inflammatory properties of MSCs, as being key mechanisms by which MSCs convert vulnerable plaques into stable plaques. In the present study, we investigated whether intravenous MSC transplantation could reduce serum inflammatory factors and minimize the regional inflammatory response allowing for stabilization of vulnerable plaque in a rabbit atherosclerotic model.

C-reactive protein (CRP/hs-CRP) is a nonspecific marker for inflammation and an independent, strong warning indicator for future coronary events in asymptomatic individuals and stable and unstable patients [15,16]. CRP has direct pro-inflammatory effects such as inducing endothelial cells to express a variety of adhesion molecules and chemotactic molecules and monocytes to synthesize and secrete potent pro-coagulant factors promoting thrombosis. In addition, TNF-α and IL-6 are also strong pro-inflammatory cytokines that play central roles in the inflammatory response and are recognized as predictive indicators of plaque instability. TNF-α and IL-6 can direct inflammatory cells to accumulate in atherosclerotic plaques, negatively impacting plaque stability and promoting thrombosis and cell necrosis [17,27]. The interaction of these factors creates an inflammatory cytokine network that synergizes to cause the development of vulnerable plaques in patients with acute coronary syndrome (ACS). To counteract such pro-inflammatory factors, IL-10, a potent anti-inflammatory factor, can inhibit inflammatory cell accumulation and proliferation of smooth muscle cells reducing tissue inflammation after injury [28]. IL-10 is minimally expressed in stable plaques, whereas its expression is elevated in vulnerable plaques.

In this study, we found that stable plaque (SP group) had relatively unchanging expression levels of hs-CRP, TNF-α, IL-6 and IL-l0. The expression of these inflammatory cytokines significantly increased in the presence of vulnerable plaque (VP group), which is consistent with previous studies. Numerous studies have found that hs-CRP, TNF-α and IL-6 as well as other inflammatory factors present in serum were significantly increased in patients with ACS and animal models with vulnerable plaque [29,30]. IL-10 expression in the SP group was significantly lower than the VP group from the third day to the fourth week post-induction. As the body's inflammatory response increased, IL-10 was synthetized and released relatively slowly to provide increasing feedback regulation.

Interestingly, we found that the levels of hs-CRP, TNF-α and IL-6 were significantly lower in the MSC group than the VP group, while IL-10 levels were greater than the VP group. Although MSC transplantation can reduce serum levels of inflammatory cytokines compared to the VP model, they were still significantly higher than that of the SP group at many time points after MSC transplantation. Similarly, Guo *et al*. found that after myocardial infarction, MSC transplantation suppressed both protein and mRNA expression of TNF-α and IL-6 in the myocardial infarction area, reduced myocardial infarction range and improved left ventricular function [7]. Moreover, Oritiz *et al*. demonstrated that after lung injury, intravenously transplanted MSCs significantly inhibited IL-1 and TNF-α production, reduced lung inflammation and continued to prevent pulmonary fibrosis [9]. More specifically, we have observed different degrees of reduction in inflammatory cytokines at various time points post-MSC transplantation. The degree of reduction at 1 week was significantly higher than that at 2 weeks, while the peak reduction occurred at 3 days. This might be because the inflammatory environment surrounding the plaque could potentially result in damage and even death of some fraction of the transplanted MSCs leading to less inhibition of pro-inflammatory cytokine production as time elapsed.

To identify the effects of MSCs on vulnerable plaques, we examined the morphological structure of plaques from the SP, VP and MSC groups using H&E and Masson’s staining techniques. Plaque in the SP group was in a stable state with a thick fibrous cap, few inflammatory cells, many smooth muscle cells, collagen and elastic fibers and no ruptured plaque. In contrast, the VP group plaques displayed more unstable characteristics including large lipid cores, a thin fibrous cap, few smooth muscle cells and fibers and more inflammatory cells. After MSC transplantation, the plaques showed more stability compared to the VP group with a thick fibrous cap, few inflammatory cells, many smooth muscle cells and fibers and no ruptured plaque or thrombosis. The plaque fibrous cap/lipid ratio in the MSC and SP groups were significantly higher than that of the VP group. This also confirmed the interpretation that intravenous MSC could stabilize vulnerable plaque and transition them into more stable states and reduce the likelihood of plaque rupture and occlusion.

We went on to measure the levels of NF-κB in plaque, which is a key transcription factor that regulates the expression of a variety of inflammatory cytokines at the gene level. We found that NF-κB expression was clearly increased in plaque from the VP group. After MSC transplantation, NF-κB expression significantly decreased, which suggests that MSCs can inhibit the expression and activation of NF-κB through presently unknown mechanisms. This could potentially lead to suppression of the local inflammatory reaction, thereby stabilizing vulnerable plaques. These findings are consistent with many other studies that have shown that that MSCs can inhibit the expression and activity of NF-κB [31–33].

Matrix metalloproteinases (MMPs) are a family of proteolytic enzymes that are important in matrix turnover and are well recognized for their roles in tissue remodeling in cardiac and atherosclerotic diseases. Previous studies have shown that the expression and activity of MMPs is closely correlated with the stability of atherosclerosis plaque, and it is thought that dysregulation of MMP enzymatic activity may be involved in many inflammatory diseases [34]. We also found that MMPs expression was higher in the VP group than the SP group. Numerous studies have shown that MSCs can inhibit the expression and enzymatic activity of MMPs. Dixon *et al*. found that MSC transplantation inhibited ventricular remodeling by reducing the synthesis of MMPs after myocardial infarction [35]. Oh *et al*. found that local application of MSCs reduced the expression of MMP-2 in a cornea injury model and prohibited angiogenesis [8]; Lozito *et al*. found that under inflammatory or hypoxic conditions, MSCs inhibited endogenous and exogenous MMP-2 and MMP-9 [36]. Consistent with past reports, we found that the expression of MMP-1, MMP-2 and MMP-9 in plaque is reduced after MSC transplantation compared to the VP group. The present study demonstrates that alterations in MMPs could alter the extracellular matrix and in turn influence the stability of the plaques. These findings suggest that MSCs may reduce regional collagen degradation to attenuate plaque vulnerability by reducing the synthesis and expression of MMPs.

The presence of apoptotic cells is one of the major features of atherosclerotic plaques [37,38]. Recent studies have shown that apoptosis of vascular endothelial cells, vascular smooth muscle cells and macrophages is involved in the formation, development, and rupture of atherosclerotic plaques [39]. As expected, inhibiting apoptosis helped to stabilize vulnerable plaques [40]. MSCs have been shown to have an anti-apoptotic role in many diseases. Wang *et al*. found that venous transplantation of MSCs reduced neuronal apoptosis and enhanced neuroprotection in rats with intracerebral hemorrhage [41]. Yeung *et al*. showed that MSCs inhibited pancreatic islet cell apoptosis [42]. In the present study, apoptotic cells were seen in the plaques of all three groups, and apoptotic cells were mainly distributed in the lipid core and tunica media in the MSC and VP groups. Apoptotic cells in the SP group were mainly distributed in the tunica media with few cells present in the lipid core and intima. Moreover, the percentage of TUNEL+ cells was significantly greater in the VP group compared to the SP group, which further supports the notion that apoptosis plays an important role in vulnerable plaque. After MSC transplantation, apoptotic cells in each region of the plaques were significantly reduced, which indicates that MSCs can reduce cell apoptosis in plaque and consequently stabilize vulnerable plaques. The mechanism by which MSCs can reduce apoptosis requires further investigation, but it is conceivable that the anti-inflammatory properties of MSCs contribute to this phenomenon.

The documented anti-inflammatory effects of MSCs are believed to be the result of MSC activation, which leads to the production and release of anti-inflammatory factors and modulation of target molecules including inducible nitric oxide synthase, indoleamine 2,3-dioxygenase, prostaglandin E2 and tumor necrosis factor alpha-stimulated gene-6 (TSG-6) [43]. Although all of these target molecules play roles in the inflammatory process, TSG-6 is particularly interesting due to the many anti-inflammatory effects regulated by this protein [44,45]. TSG-6 is not constitutively expressed in normal tissues or cells, but rather is up-regulated in response to pro-inflammatory cytokines such as TNF-α, IL-1, and IL-6 [46,47]. It serves as a feedback mechanism to inhibit inflammation-mediated extracellular matrix remodeling by reducing the expression of inflammatory factors, inhibition of neutrophil infiltration and plasmin activity [48,49]. These anti-inflammatory effects have been well characterized in several animal models [22,50,51]. Mechanistically, TSG-6 can reduce the production of pro-inflammatory cytokines through suppression of NF-κB signaling, which initiates a cascade of pro-inflammatory cytokines [52]. Transplanted MSCs played a crucial role in suppressing local inflammation and fibrosis in models of myocardial infarction, corneal damage and more recently peritoneal injury, and these anti-inflammatory effects may be attributable to the secretion of TSG-6 by MSCs [22,53,54]. In the present study, we measured the mRNA expression of TSG-6 in atherosclerotic plaques of all three groups using realtime-PCR and Western blot. Our data demonstrate that mRNA and protein expression of TSG6 increased in the VP group compared to the SP group and further increased after MSC transplantation. Histological and immunohistochemical results indicated that inflammation was activated in the atherosclerotic plaque after liquid nitrogen induction, NF-κB and other pro-inflammatory factors were increasingly expressed, TSG-6 may have had less increase by feedback mechanisms, and the serum results illustrate the same phenomenon. After MSC transplantation, TSG-6 showed greater expression compared with the SP and VP groups, which indicated TSG-6 may an important anti-inflammatory agent secreted by MSC for the reduction of regional inflammation and improve plaque stabilization

Overall, our results suggest that transplanted MSCs have anti-inflammatory properties including enhanced expression of anti-inflammatory factors such as TSG-6 and Il-10 which may down-regulate the NF-κB signaling pathway, expression of pro-inflammatory factors and reduce infiltration of neutrophils and macrophages into plaques. Therefore, MSCs likely play an important role in inhibiting inflammation and stabilizing vulnerable plaques.

## Supporting Information

S1 ARRIVE Checklist NC3Rs ARRIVE Guidelines Checklist. (PDF)

S1 File The tables behind charts. Hs-CRP levels in three groups at different time points (Table A). TNF-α levels in three groups at different time points (Table B). IL-6 levels in three groups at different time points (Table C). IL-10 levels in three groups at different time points (Table D). Cap/core ratio of the plaques in different groups (Table E). Levels of NF-κB, MMPs and TIMP-1 expression in plaque tissue (Table F). The apoptosis index in different groups after 4 week (Table G). The mRNA and protein expressions of TSG6 in different groups after 4 week (Table H).
(DOC)

S2 File Raw data in the paper. (XLS)
