# Supplementary material for: Mesenchymal Stem Cells Stabilize Atherosclerotic Vulnerable Plaque by Anti-Inflammatory Properties
Source: PLoS One. 2015 Aug 19;10(8):e0136026. doi: 10.1371/journal.pone.0136026 (PMC4546153; doi:10.1371/journal.pone.0136026)
Supplement: S1 File — Hs-CRP levels in three groups at different time points (Table A). TNF-α levels in three groups at different time points (Table B). IL-6 levels in three groups at different time points (Table C). IL-10 levels in three groups at different time points (Table D). Cap/core ratio of the plaques in different groups (Table E). Levels of NF-κB, MMPs and TIMP-1 expression in plaque tissue (Table F). The apoptosis index in different groups after 4 week (Table G). The mRNA and protein expressions of TSG6 in different groups after 4 week (Table H). (DOC) [file pone.0136026.s002.doc]

Table A. Hs-CRP levels in three groups at different time points (ng/ml)

| group |  | Time | | | | | | Sum | F | *P* |
| --- | --- | --- | --- | --- | --- | --- | --- | --- | --- | --- |
|  |  | D1 | D2 | D3 | D7 | D14 | D28 |  |  |  |
| MSC  （10） | 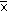 | 17.96 | 22.92a | 25.20a | 21.92 | 19.25c | 17.36bc | 20.769 | 4.703 | 0.002 |
|  | s | 2.61 | 4.67 | 4.85 | 5.10 | 5.07 | 5.22 | 5.296 |  |  |
| VP  （8） | 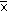 | 29.89* | 43.11*a | 63.82*ab | 51.84*a | 50.92*a | 31.07*bcde | 45.107* | 13.941 | <0.001 |
|  | s | 7.58 | 10.25 | 12.52 | 8.40 | 11.6 | 9.55 | 15.439 |  |  |
| SP  （10） | 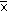 | 7.00*# | 6.69*# | 7.41*# | 6.97*# | 7.35*# | 7.26*# | 7.112*# | 0.350 | 0.880 |
|  | s | 1.52 | 1.51 | 1.75 | 1.31 | 1.20 | 1.24 | 1.394 |  |  |
| Sum | 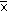 | 17.45 | 22.89a | 29.88ab | 25.13ac | 24.05ac | 17.67bcde | 22.845 | 21.670 | <0.001 |
|  | s | 10.22 | 15.93 | 24.21 | 19.10 | 19.23 | 11.25 | 17.624 |  |  |
| F |  | 88.660 | 94.839 | 126.789 | 139.404 | 75.878 | 38.850 | 428.245 | （F=11.857  *P*<0.001） | |
| *P* |  | <0.001 | <0.001 | <0.001 | <0.001 | <0.001 | <0.001 | <0.001 |  |  |

* Compared with MSC, P<0.05; # compared with VP, P<0.05; a compared with D1, P<0.05; b compared with D2, P<0.05; c compared with D3, P<0.05; d compared with D7, P<0.05; e compared with D14, P<0.05.

Table B. TNF-α levels in three groups at different time points (ng/ml)

| group |  | Time | | | | | | Sum | F | *P* |
| --- | --- | --- | --- | --- | --- | --- | --- | --- | --- | --- |
|  |  | D1 | D2 | D3 | D7 | D14 | D28 |  |  |  |
| MSC  （10） | 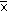 | 9.83 | 10.96 | 11.95 | 9.59c | 8.83c | 8.13bc | 9.879 | 3.647 | 0.007 |
|  | s | 2.09 | 2.45 | 2.69 | 2.23 | 2.24 | 2.41 | 2.595 |  |  |
| VP  （8） | 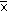 | 13.84* | 16.62*a | 20.16*ab | 25.71*abc | 22.30*ab | 18.42*ad | 19.505 | 11.429 | <0.001 |
|  | s | 2.31 | 2.54 | 4.01 | 4.64 | 4.13 | 3.5 | 5.173 |  |  |
| SP  （10） | 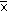 | 7.83*# | 8.36*# | 8.00*# | 7.37# | 7.27# | 7.34# | 7.71 | 0.652 | 0.662 |
|  | s | 1.38 | 1.80 | 1.97 | 1.97 | 1.77 | 1.69 | 1.748 |  |  |
| Sum | 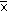 | 10.26 | 11.65 | 12.91 | 13.40 | 12.12 | 10.79 | 11.855 | 8.056 | <0.001 |
|  | s | 3.09 | 4.03 | 5.69 | 8.50 | 7.11 | 5.51 | 5.95 |  |  |
| F |  | 21.689 | 30.181 | 30.715 | 52.115 | 44.213 | 50.074 | 245.006 | （F=9.362  *P*<0.001） | |
| *P* |  | <0.001 | <0.001 | <0.001 | <0.001 | <0.001 | <0.001 | <0.001 |  |  |

* Compared with MSC, P<0.05; # compared with VP, P<0.05; a compared with D1, P<0.05; b compared with D2, P<0.05; c compared with D3, P<0.05; d compared with D7, P<0.05; e compared with D14, P<0.05.

Table C. IL-6 levels in three groups at different time points (pg/ml)

| group |  | Time | | | | | | Sum | F | *P* |
| --- | --- | --- | --- | --- | --- | --- | --- | --- | --- | --- |
|  |  | D1 | D2 | D3 | D7 | D14 | D28 |  |  |  |
| MSC | 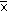 | 81.21 | 79.43 | 85.08 | 95.44b | 87.84 | 81.87d | 85.144 | 1.723 | 0.149 |
| （10） | s | 11.96 | 12.57 | 13.43 | 17.87 | 15.92 | 12.83 | 14.666 |  |  |
| VP | 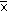 | 86.20 | 95.81* | 102.45*a | 121.88*abc | 108.28*a | 92.93d | 101.258 | 5.791 | 0.001 |
| （8） | s | 12.47 | 15.53 | 12.51 | 19.76 | 16.72 | 15.14 | 18.783 |  |  |
| SP | 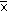 | 68.80*# | 76.48# | 74.489# | 74.57*# | 68.62*# | 72.86# | 72.632 | 0.656 | 0.658 |
| （10） | s | 9.97 | 11.58 | 13.1 | 13.14 | 13.90 | 12.53 | 12.285 |  |  |
| Sum | 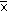 | 78.20 | 83.05 | 86.26 | 95.54 | 86.813 | 81.81 | 85.280 | 5.852 | <0.001 |
|  | s | 13.27 | 15.13 | 16.98 | 25.17 | 21.9 | 15.27 | 18.959 |  |  |
| F |  | 5.680 | 5.404 | 10.204 | 17.364 | 14.659 | 4.974 | 49.492 | （F=1.951  *P*=0.044） | |
| *P* |  | 0.009 | 0.011 | 0.001 | <0.001 | <0.001 | 0.000 | 0.015 |  |  |

* Compared with MSC, P<0.05; # compared with VP, P<0.05; a compared with D1, P<0.05; b compared with D2, P<0.05; c compared with D3, P<0.05; d compared with D7, P<0.05; e compared with D14, P<0.05.

Table D. IL-10 levels in three groups at different time points (ng/ml)

| group |  | Time | | | | | | Sum | F | *P* |
| --- | --- | --- | --- | --- | --- | --- | --- | --- | --- | --- |
|  |  | D1 | D2 | D3 | D7 | D14 | D28 |  |  |  |
| MSC | 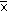 | 12.24 | 15.87a | 20.93ab | 26.67abc | 24.56abc | 22.35abd | 20.437 | 27.246 | <0.001 |
| （10） | s | 2.02 | 2.38 | 3.44 | 3.96 | 4.38 | 3.38 | 5.948 |  |  |
| VP | 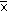 | 10.50* | 10.36* | 12.30*b | 14.91*ab | 13.30*abd | 12.48*abd | 12.309 | 7.193 | <0.001 |
| （8） | s | 1.44 | 1.67 | 2.13 | 2.79 | 1.88 | 1.56 | 2.443 |  |  |
| SP | 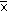 | 9.48* | 9.34* | 9.29*# | 10.14*# | 9.64*# | 9.39*# | 9.545 | 0.370 | 0.867 |
| （10） | s | 1.67 | 1.68 | 1.43 | 1.80 | 1.62 | 1.67 | 1.605 |  |  |
| Sum | 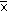 | 10.76 | 11.97 | 14.31 | 17.40 | 16.01 | 14.90 | 14.225 | 28.241 | <0.001 |
|  | s | 2.07 | 3.54 | 5.71 | 7.84 | 7.24 | 6.24 | 6.154 |  |  |
| F |  | 6.378 | 31.497 | 47.838 | 70.444 | 51.391 | 78.123 | 244.606 | （F=12.684  *P*<0.001） | |
| *P* |  | 0.006 | <0.001 | <0.001 | <0.001 | <0.001 | <0.001 | <0.001 |  |  |

* Compared with MSC, P<0.05; # compared with VP, P<0.05; a compared with D1, P<0.05; b compared with D2, P<0.05; c compared with D3, P<0.05; d compared with D7, P<0.05; e compared with D14, P<0.05.

Table E. Cap/core ratio of the plaques in different groups

| Group | Cap/core ratio |
| --- | --- |
| MSC（n=10） | 0.219±0.027 |
| VP（n=8） | 0.153±0.018^*^ |
| SP（n=10） | 0.238±0.040^#^ |
| F | 28.298 |
| *P* | <0.001 |

* Compared with MSC, P<0.01; # compared with VP, P<0.01

Table F. Levels of NF-κB, MMPs and TIMP-1 expression in plaque tissue

| Group | NF-κB | | MMP-1 | | MMP-2 | | MMP-9 | |
| --- | --- | --- | --- | --- | --- | --- | --- | --- |
| MSCs(10) | | 176.44±25.50 | | 124.44±28.85 | | 138.79±23.77 | | 190.29±26.00 |
| VP(8) | 274.90±41.93^**^ | | 215.27±33.28^**^ | | 213.83±36.10^**^ | | 252.04±32.81^**^ | |
| SP(10) | 131.64±29.52^**##^ | | 93.63±19.33^*##^ | | 114.27±17.51^##^ | | 150.77±28.84^**##^ | |
| F | 44.953 | | 46.530 | | 24.909 | | 27.108 | |
| *P* | <0.001 | | <0.001 | | <0.001 | | <0.001 | |

Compared with MSC, *P<0.05, ** P＜0.01; compared with VP, #P<0.05, ## P＜0.01.

Table G. The apoptosis index in different groups after 4 week (
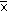
±s)

| Group | apoptosis index（AI） |
| --- | --- |
| MSC（n=6） | 45.17±9.66 |
| VP（n=6） | 68.00±12.05^*^ |
| SP（n=6） | 32.67±5.85^*#^ |
| F | 21.178 |
| *P* | <0.001 |

Compared with MSC, * P＜0.05; compared with VP, # P＜0.05.

Table H. The mRNA and protein expressions of TSG6 in different groups after 4 week (
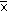
±s)

| Group | TSG6 mRNA relative expression | TSG6 protein relative expression |
| --- | --- | --- |
| Group MSC（n=6） | 7.89±3.08 | 0.747±0.119 |
| Group VP（n=6） | 2.27±0.18^*^ | 0.429±0.089^**^ |
| Group SP（n=6） | 1.01±0.12^**##^ | 0.213±0.027^**#^ |
| F | 106.082 | 28.681 |
| *P* | <0.001 | <0.05 |

Compared with MSC, *P<0.05, ** P＜0.01; compared with VP, #P<0.05, ## P＜0.01.
